# Deep oxidative desulfurization of gas oil by iron(III)-substituted polyoxometalate immobilized on nickel(II) oxide, ((n-C_4_H_9_)_4_N)_4_H[PW_11_FeO_39_]@NiO, as an efficient nanocatalyst

**DOI:** 10.1038/s41598-023-42545-9

**Published:** 2023-09-14

**Authors:** Mohammad Ali Rezvani, Kolsom Ghasemi, Hadi Hassani Ardeshiri, Masomeh Aghmasheh

**Affiliations:** 1 Department of Chemistry, Faculty of Science, University of Zanjan, Zanjan, 451561319 Iran; 2 Catalysts and Organic Synthesis Research Laboratory, Department of Chemistry, Iran University of Science and Technology, Tehran, 16846-13114 Iran

**Keywords:** Catalysis, Environmental chemistry, Inorganic chemistry, Chemical synthesis

## Abstract

Sulfur compounds are among the most unfavorable constituents of petroleum derivatives, so stringent regulations have been established to curb their atmospheric emissions. In this regard, a new nanocomposite ((n-C_4_H_9_)_4_N)_4_H[PW_11_FeO_39_]@NiO) was synthesized composed of quaternary ammonium bromide salt of iron^III^-substituted Keggin-type polyoxometalate immobilized on nickel(II) oxide nanoceramics via sol–gel method. The assembled (n-C_4_H_9_)_4_N)_4_H[PW_11_FeO_39_]@NiO nanocomposite was identified by FT-IR, UV–Vis, XRD, SEM, EDX, and TGA-DTG methods. The characterization results exhibited that ((n-C_4_H_9_)_4_N)_4_H[PW_11_FeO_39_] dispersed uniformly over the surface of the NiO nanoceramics. The ((n-C_4_H_9_)_4_N)_4_H[PW_11_FeO_39_]@NiO nanocomposite was employed as a heterogeneous nanocatalyst in the extractive coupled oxidation desulfurization (ECOD) of real gas oil and dibenzothiophene (DBT) as a model compound. Under relatively moderate conditions, the catalytic performance of the ((n-C_4_H_9_)_4_N)_4_H[PW_11_FeO_39_]@NiO in the ECOD procedure was studied by incorporating acetic acid/hydrogen peroxide as an oxidant system at a volume ratio of 1:2. According to the ECOD results, the ((n-C_4_H_9_)_4_N)_4_H[PW_11_FeO_39_]@NiO demonstrated the effectiveness of up to 95% with 0.1 g at 60 °C under optimal operating conditions. Moreover, the ((n-C_4_H_9_)_4_N)_4_H[PW_11_FeO_39_]@NiO nanocatalyst could be separated and reused for five runs without a noticeable decrease in the ECOD process. This study provides a promising way to meet the target of ultra-low sulfur as an essential process in oil refineries.

## Introduction

Polyoxometalates (POMs) are a particular type of inorganic metal-oxo clusters with well-defined topological architecture^1,2^. Generally, POMs are constructed by primary high-valent d-block transition metals such as Mo, W, Nb, Ta, and V^3^. Various structural classes, including Keggin [{XM_12_O_40_}^3/4−^] (X = P, Si, B and M = and M = Mo, W)^4^, Anderson [{AM_6_O_24_}^9/10−^] (A = Mn, Fe, Cr, Al, I, etc. and M = Mo, W)^5^, Wells–Dawson [{X_2_M_18_O_62_}^6−^]^6^, (X = P, Si and M = Mo, W), Evans–Showell [{Co_2_Mo_10_H_4_O_38_}^6−^]^7^, etc. were employed as construction units to build polyoxometalates-based platform. Among the different structures, Keggin-type polyoxometalates have attracted the attention of researchers due to their unique structure and characteristics, such as high selectivity, redox properties, and thermal stability^8,9^. The Keggin structure is composed of a globelike cluster with corner/edge sharing polymeric WO_6_ units and a central tetrahedron (PO_4_), which was found to be a very active and selective oxidation catalyst^10,11^. Nevertheless, some challenges in industrial applications are constrained by their low surface area and poor reusability^12,13^. Hence, the immobilization of Keggin-type POMs on the surface of metal oxides as an inexpensive and eco-friendly materials is a promising approach to boost their catalytic features and mechanical strength^14^. To architect the polyoxometalate-based heterogeneous catalysts, several metal oxides, such as TiO_2_, NiO, CoFe_2_O_4_, NiZn_2_O_4_, NiCo_2_O_4,_ etc., were used as carriers^15–17^. In this research, nickel(II) oxide (NiO) was deemed to be a support for the immobilization of Keggin-type POM. Nickel(II) oxide has been extensively studied because of its non-toxicity, simple preparation, environmentally friendly, and good mechanical hardness^18^. These features make it a promising candidate for a wide variety of applications, such as green catalysis, electrochemistry, desulfurization, biology, and medicine. Recently, the production of ultra-low-sulfur fuels has become a major challenge for oil refineries with regard to environmental safety^19,20^. Sulfur compounds from petroleum derivatives (gas oil) contribute to atmospheric pollution and have significant environmental impacts^21,22^. Therefore, the sulfur compounds in the current gas oil should be considered hazardous materials. In order to minimize the adverse environmental effects of sulfur-containing compounds, various desulfurization approaches have been developed by industry and academic researchers. Hydrodesulfurization (HDS), also called hydrotreatment, is an industrial refining process that requires high-pressure (150 to 250 psig) and high temperature (200 to 425 °C) and utilizes gaseous hydrogen to decrease the sulfur concentration in petroleum fractions (particularly gas oil) to hydrogen sulfide^23^. The HDS strategy was used for a long time as an industrial refining process to remove S compounds over heterogenous catalysts. Nonetheless, taking into account the impossibility of removing heterocyclic sulfur compounds containing sulfur (DBT and its derivatives), and the requirement for demanding operating conditions (high temperature and high pressure), it is necessary to develop an effective new procedure to reduce pollution and costs substantially^24^. Therefore, alternative non-HDS technologies, including oxidative desulfurization (ODS)^25^, adsorption (ADS)^26^, extraction (EDS)^27^, and bio-desulfurization (BDS)^28^ were utilized as promising strategies for the desulfurization of real liquid fuels. Catalytic Oxidative desulfurization (ODS) is the most appropriate process for removing heterocyclic sulfur compounds from fuels under ambient pressure at below 100 °C^29,30^. In the ODS procedure, liquid hydrocarbon fuels are oxidized to sulfones/sulfoxides using a suitable oxidant. There are some oxidizers, such as ozone (O_3_), organic peroxides, Fenton's reagent, ferrate, hydrogen peroxide (H_2_O_2_), and oxygen (O_2_). H_2_O_2_ is an appropriate oxidant in the ODS process of petroleum hydrocarbon fraction (C10–C22) with boiling range of 175–375 °C (light fuel oil) because of its low price and eco-friendly^31^. In the follow-up to our earlier research, this study confirms that the introduction of iron^III^ into the Keggin-type POM unit leads to a considerable development in the catalytic ODS process. Herein, a quaternary ammonium bromide salt of Fe^III^-substituted Keggin-type phosphotungstate@Nickel(II) oxide nanocomposite, ((n-tBu)_4_N)_4_H[PW_11_FeO_39_]@NiO, was synthesized as a new phase transfer catalyst for extractive coupled oxidative desulfurization (ECOD) of prepared thiophenic heterocyclic compound (DBT) and real fuel. To assess the catalytic performance of the ((n-tBu)_4_N)_4_H[PW_11_FeO_39_]@NiO, the ECOD procedure was conducted with an acetic acid/hydrogen peroxide oxidizing agent under mild reaction conditions. The influence of nanocatalyst dosage, reaction temperature, and time were perused. Furthermore, the kinetic and recyclability of the ((n-tBu)_4_N)_4_H[PW_11_FeO_39_]@NiO nanocatalyst in the architected ECOD procedure were evaluated.

## Experimental section

### Materials and methods

The following chemicals and solvents were obtained from commercial suppliers and used as received. Acetic acid (CH_3_COOH, 99.7%), n-heptane (C_7_H_16_, 99%), hydrogen peroxide (H_2_O_2_, 30%), nickel (II) nitrate hexahydrate (Ni(NO_3_)_2_·6H_2_O, 99%), acetonitrile (MeCN, 99%), and citric acid monohydrate (C_6_H_8_O_7_·H_2_O, 99%) were bought out of Fluka. Sodium tungstate dihydrate (Na_2_WO_4_·2H_2_O, 99%), disodium hydrogen phosphate (Na_2_HPO_4_, 99%), dibenzothiophene (DBT, 98%), and tetra (n-butyl) ammonium bromide (Bu_4_N^+^Br^−^, 98%) were supplied by Merk company. The structural property of the synthesized materials was observed by the XRD method, (HTK 1200n—Bruker D8) using Cu Kα1 radiation (λ = 0.15405 nm) with a scan angle (2θ) range between 10° and 80°. A UV–Vis method was used to study the optical characteristics of the synthesized nanocomposite using a CARY 5E UV–VIS–NIR Spectrophotometer at room temperature from across the wavelength range of 190–1100 nm. Furthermore, FT-IR spectrometer (Bruker–Tensor 27) was used to record the FT-IR spectra of the synthesized samples from the range of 400–4000 cm^−1^. The material morphologies were observed by SEM analysis using a FEI-Quanta FEG 200F, along with an energy-dispersive X-ray (EDX) spectroscopy. Moreover, thermogravimetric analyses (TGA) and derivative thermal gravimetric (DTG) experiments were conducted using a NETZSCH STA 409 PC/PG Germany spectrometer. The content of total sulfur and mercaptans into real gas oil and simulated fuel were determined using X-ray fluorescence (XRF) with a TANAKA X-ray fluorescence spectrometer RX-360 SH.

### Preparation of the ((n-C_4_H_9_)_4_N)_4_H[PW_11_FeO_39_]@NiO nanocatalyst

#### Preparation of ((n-tBu)_4_N)_4_H[PW_11_FeO_39_]

The quaternary ammonium salt (Bu_4_N^+^Br^−^) of Keggin-type phosphotungstoferrate, ((n-C_4_H_9_)_4_N)_4_H[PW_11_FeO_39_], was prepared based on the literature^32^. The requisite amount of Na_2_WO_4_·2H_2_O (3.29 g) was dissolved in 25 mL of distilled water (DW) while stirring magnetically. Afterward, the prepared solution from 0.15 g of Na_2_HPO_4_ and 0.37 g Ni(NO_3_)_2_·6H_2_O were added drop-wise to the solution of Na_2_WO_4_·2H_2_O. Then, the pH value for the resultant mixture was adjusted to 4.5 by agitation, and the blend was heated to the temperature of 80–85 °C. An aqueous solution prepared from 1.45 g of Bu_4_N^+^Br^−^ was added gradually to the above mixture to engender a creamy white precipitation of ((n-C_4_H_9_)_4_N)_4_H[PW_11_FeO_39_]. The resultant precipitate was separated by filtration, washed with ethanol, and dried in conventional conditions.

#### Preparation of NiO nanoceramics

The preparation of the nickel(II) oxide nanoceramics is as follows: 1.90 g of citric acid monohydrate has been dissolved into 20 mL of DW. The resulting solution was added gradually to the aqueous solution of Ni(NO_3_)_2_·6H_2_O (2.90 g in 20 mL of DW) and magnetically stirred at 80 °C for 60 min to create a green gel. Eventually, the resultant green gel was dried at 80 °C for 1 h, and then calcined at 400 °C for 4 h.

#### Preparation of ((n-C_4_H_9_)_4_N)4H[PW_11_FeO_39_]@NiO nanocomposite

**Figure 1:**
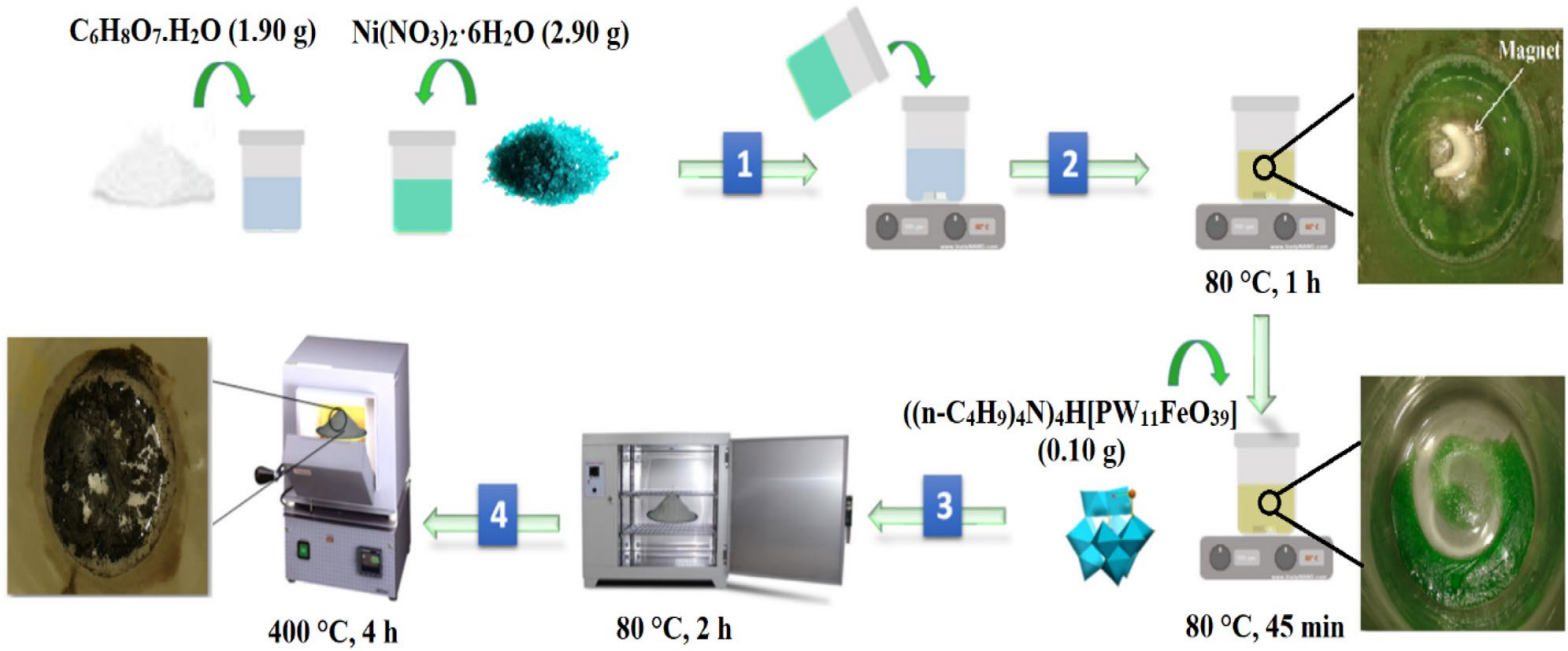
Synthesis schematic of the ((n-C_4_H_9_)_4_N)_4_H[PW_11_FeO_39_]@NiO nanocomposite.

An outline of the ((n-C_4_H_9_)_4_N)_4_H[PW_11_FeO_39_]@NiO synthesis process is illustrated in Fig. 1. 0.10 g of the ((n-C_4_H_9_)_4_N)_4_H[PW_11_FeO_39_] was dissolved in 20 mL DW using heat and dispersion. The obtained solution was added drop-wise to the prepared green gel from NiO nanoceramics. The blended mixture was heated to 80 °C and agitated strenuously for 45 min. Eventually, the obtained green gel was dried at 80 °C for 2 h and calcined at 400 °C for 4 h.

### ECOD process of model/real gas oil

**Figure 2:**
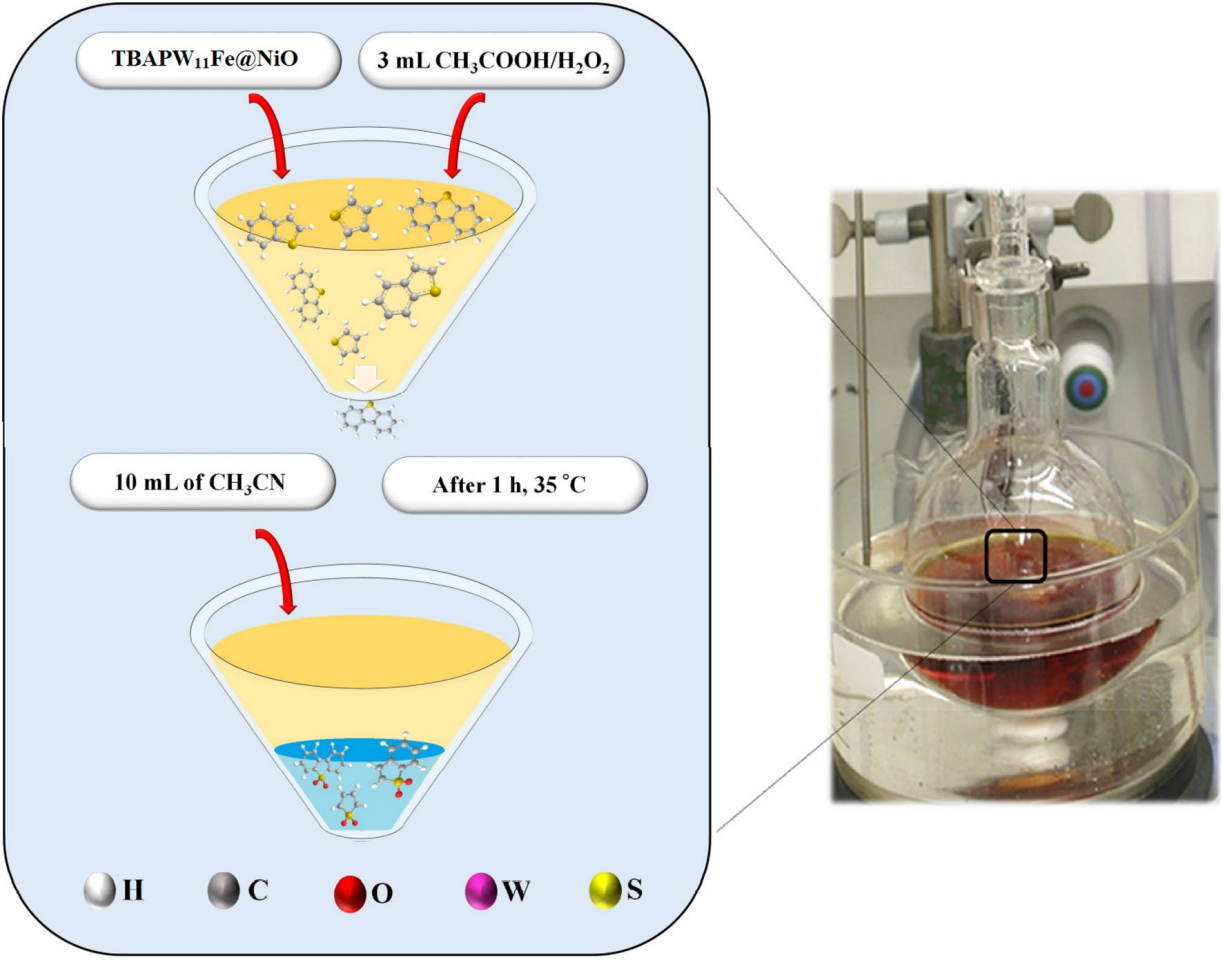
An outline of the ECOD process. (The ECOD process of real and model gas oil was carried out using the ((n-C_4_H_9_)_4_N)_4_H[PW_11_FeO_39_]@NiO nanocatalyst by acetic acid/H_2_O_2_ oxidizing system. After the ECOD procedure, the treated thiophenic model fuels and real gas oil were cooled to room temperature, and 10 mL of MeCN was added to extract the RSO (sulfoxide) and RSO_2_ (sulfones) from the gas oil phase to the water phase).

The ECOD process involves two steps: oxidation, followed by extraction. Oxidant, catalyst, and operating conditions play an essential role in the ECOD process. In this study, a certain amount of the heterocyclic sulfur compound containing DBT was dissolved in n-heptane (500 ppmw S content) as a thiophenic model gas oil to investigate the catalytic performance of the ((n-C_4_H_9_)_4_N)_4_H[PW_11_FeO_39_]@NiO in the extractive-oxidative desulfurization procedure. For this purpose, 50 mL of each HSC was added to the round-bottom flask placed in the water bath. The water bath was warmed up to 25, 30, 35, and 40 °C in distinct experiments. Afterward, a combination of CH_3_COOH/H_2_O_2_ (v/v ratio of ½) and the corresponding amount of the ((n-C_4_H_9_)_4_N)_4_H[PW_11_FeO_39_]@NiO nanocatalyst (0.02–0.12 g) were added to the reaction vessel while stirring vigorously. After 60 min, the resulting solution was subsequently cooled to room temperature after the ECOD process, and 10 mL of MeCN as an extraction solvent was added to remove the oxidative products. Finally, the concentration of total sulfur (wt%) and mercaptan (ppm) in the prepared gas oil was determined before and after the reaction based on the standard tests (D-4294 and D-3227). The DBT removal yield was calculated according to the formula below:

1 $${\text{Sulfur \, removal \, efficiency }}(\% ) = \left[ {1 - \frac{{S_{f} }}{{S_{i} }}} \right] \times 100$$

where *S*_*i*_ and *S*_*f*_ report the initial and residual sulfur content after the ECOD into *n-*heptane. The ECOD of real gas oil was carried out in the same manner as the prepared thiophenic heterocyclic compound (DBT) was desulfurized. The schematic of the ECOD process is illustrated in Fig. 2.

## Results and discussion

### Characterization of materials

**Figure 3:**
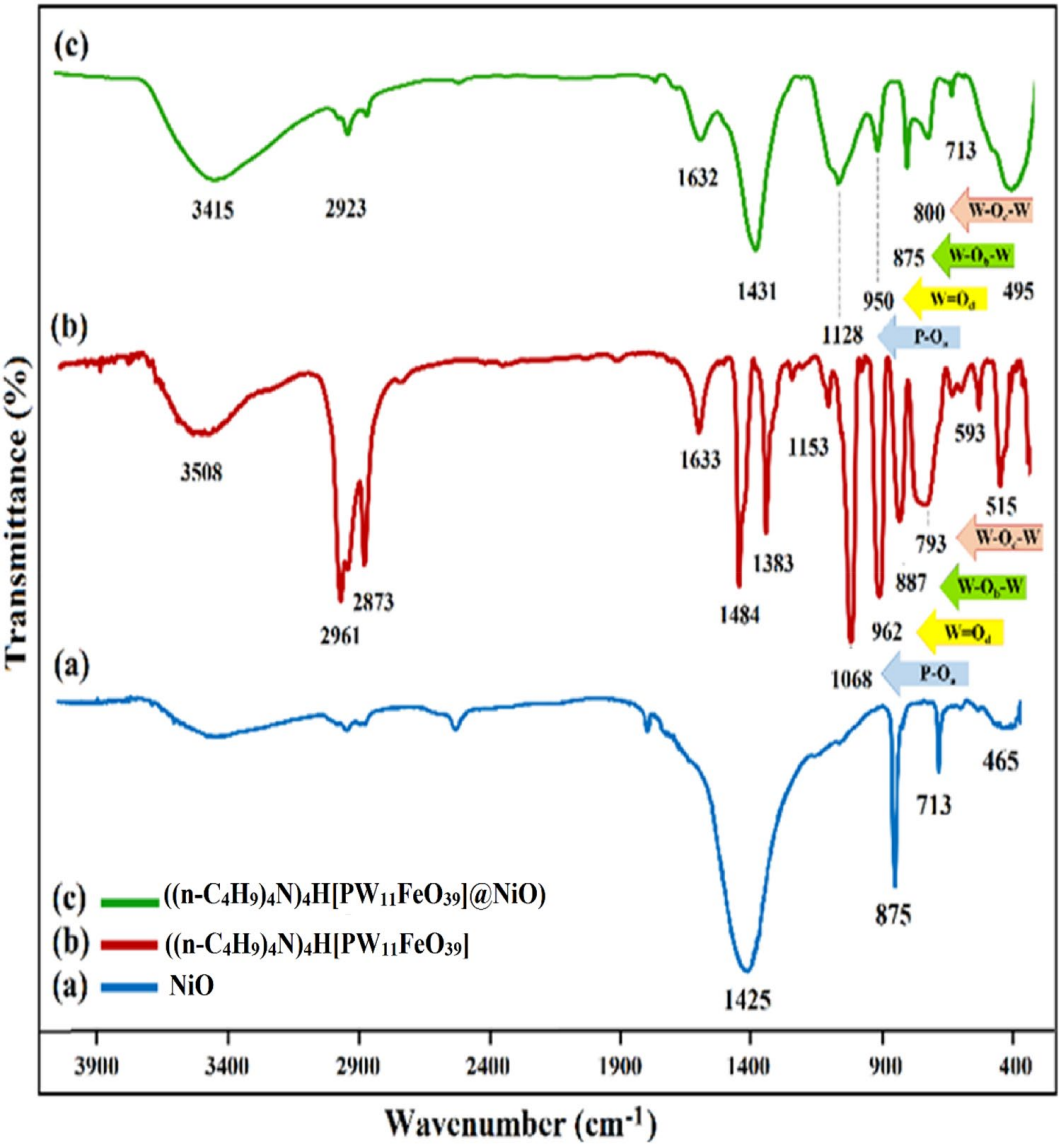
FT-IR spectra of (a) NiO nanoparticles, (b) ((n-C_4_H_9_)_4_N)_4_H[PW_11_FeO_39_], and (c) ((n-C_4_H_9_)_4_N)_4_H[PW_11_FeO_39_]@NiO.

The ((n-C_4_H_9_)_4_N)_4_H[PW_11_FeO_39_]@NiO nanocomposite was synthesized via the sol–gel method. The immobilization of ((n-C_4_H_9_)_4_N)_4_H[PW_11_FeO_39_] on NiO nanoceramic was confirmed by several analyses. FT-IR spectroscopy was used to determine the structure and functional groups of the materials in the region of 400–4000 cm^−1^. The characteristic FT-IR spectra of (a) NiO nanoparticles, (b) ((n-C_4_H_9_)_4_N)_4_H[PW_11_FeO_39_], and (c) ((n-C_4_H_9_)_4_N)_4_H[PW_11_FeO_39_]@NiO were displayed in Fig. 3 perspicuously. According to the obtained infrared spectrum for NiO (Fig. 3a), the broad peak at 465 cm^−1^ is attributed to the stretching vibrations of Ni–O groups^33^. This spectrum does not exhibit characteristic bands of impurities or other precursor compounds. Furthermore, the stretching vibration mode at 713 cm^−1^ is assigned to the Ni–O–H groups. The typical peaks at 1068, 962, 887, and 793 cm^−1^ were caused by the stretching vibrations of phosphotungstoferrate anions ((n-C_4_H_9_)_4_N)_4_H[PW_11_FeO_39_]) implying P–Oa, terminal W = Od, corner-sharing W–Ob–W, and edge-sharing W–Oc–W bonds, respectively (Fig. 3b)^34,35^. In addition, the characteristic peaks at 1484 and 1383 cm^−1^ correspond to scissor vibrations of N^+^-CH_3_, which are attributed to the tetra (n-butyl) ammonium bromide salt of iron^III^-substituted Keggin-type phosphotungstate^36^. As shown in Fig. 3c, the peaks corresponding to ((n-C_4_H_9_)_4_N)_4_H[PW_11_FeO_39_] in FT-IR spectrum of the product (((n-C_4_H_9_)_4_N)_4_H[PW_11_FeO_39_]@NiO) were observed with slight shifts (793 → 800, 887 → 875, 962 → 950, 1068 → 1128), which indicates that the prepared nanocomposite has maintained its structure after immobilization on NiO nanoparticles. Moreover, the characteristic peaks of NiO nanoparticles were observed with some coverage by the ((n-C_4_H_9_)_4_N)_4_H[PW_11_FeO_39_], confirming that the nanocomposite has been successfully synthesized.

**Figure 4:**
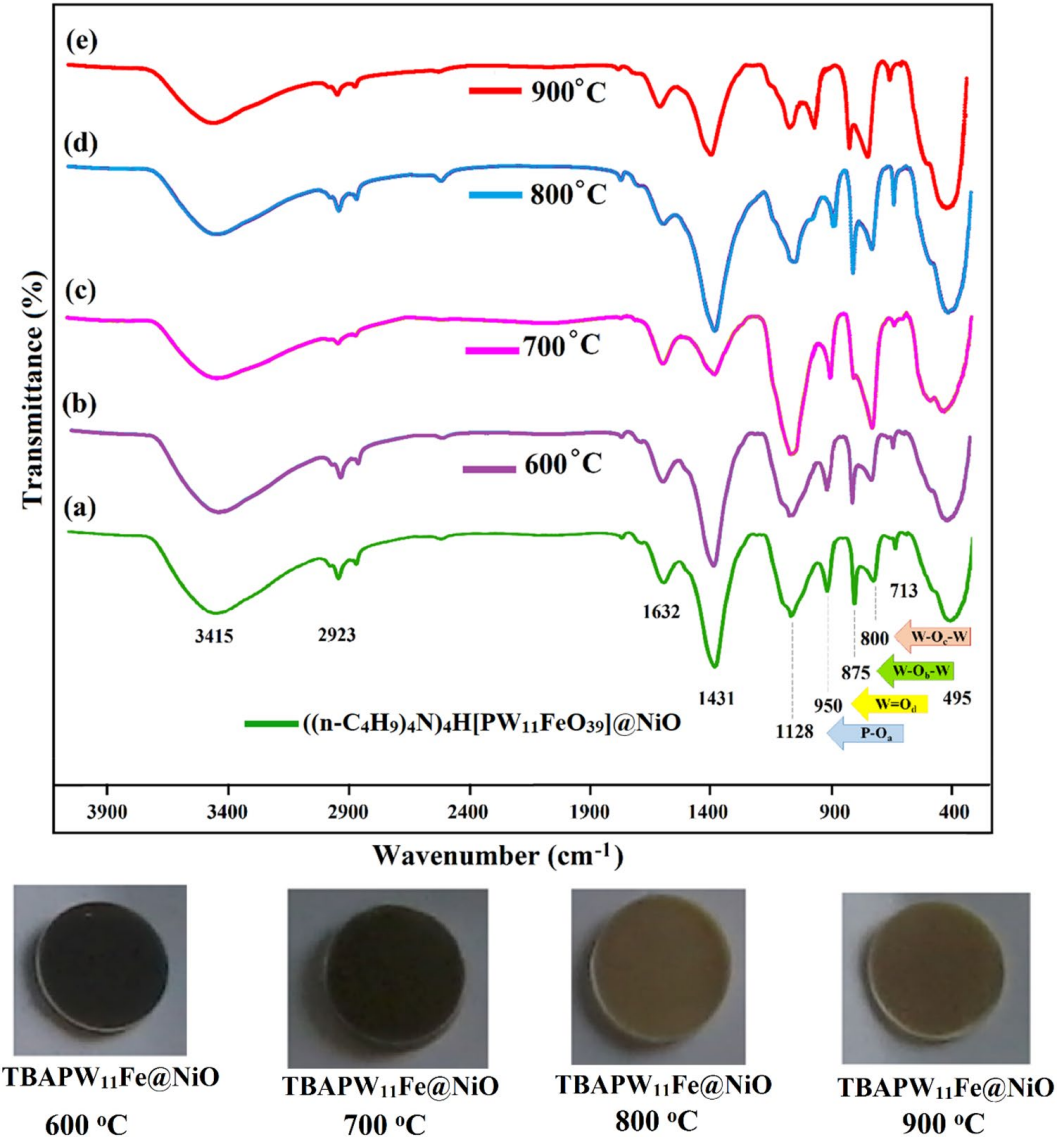
Thermal FT-IR spectra of the ((n-C_4_H_9_)_4_N)_4_H[PW_11_FeO_39_]@NiO nanocomposite at different temperatures of 600 °C, 700 °C, 800 °C, and 900 °C with the corresponding KBr pellets.

For a deeper understanding, thermal FT-IR spectra of the ((n-C_4_H_9_)_4_N)_4_H[PW_11_FeO_39_]@NiO nanocomposite at different temperatures of 600 °C, 700 °C, 800 °C, and 900 °C are displayed in Fig. 4 with the corresponding KBr pellets. With this analysis, the reaction mechanisms of the sol–gel process can be specified. At lower temperatures, the FT-IR spectrum exhibits a high degree of complexity, whereas at higher temperatures, some of absorption bands diminish, resulting in a simpler spectrum. The absorption band at 3415 cm^−1^ has been attributed to the stretching vibration of the hydroxyl group in water molecules, thus providing confirmation of the presence of water within the nanocomposite structure. As the calcination temperature increases, there is a decrease in the peak related to the O–H group, but this band persisted up to 900 °C. The reduction in intensity observed at lower temperatures can be attributed to the elimination of water from the nanocomposite. Conversely, the retention of intensity at higher temperatures may be attributed to the adsorption of moisture during the standard preparation method for samples.

**Figure 5:**
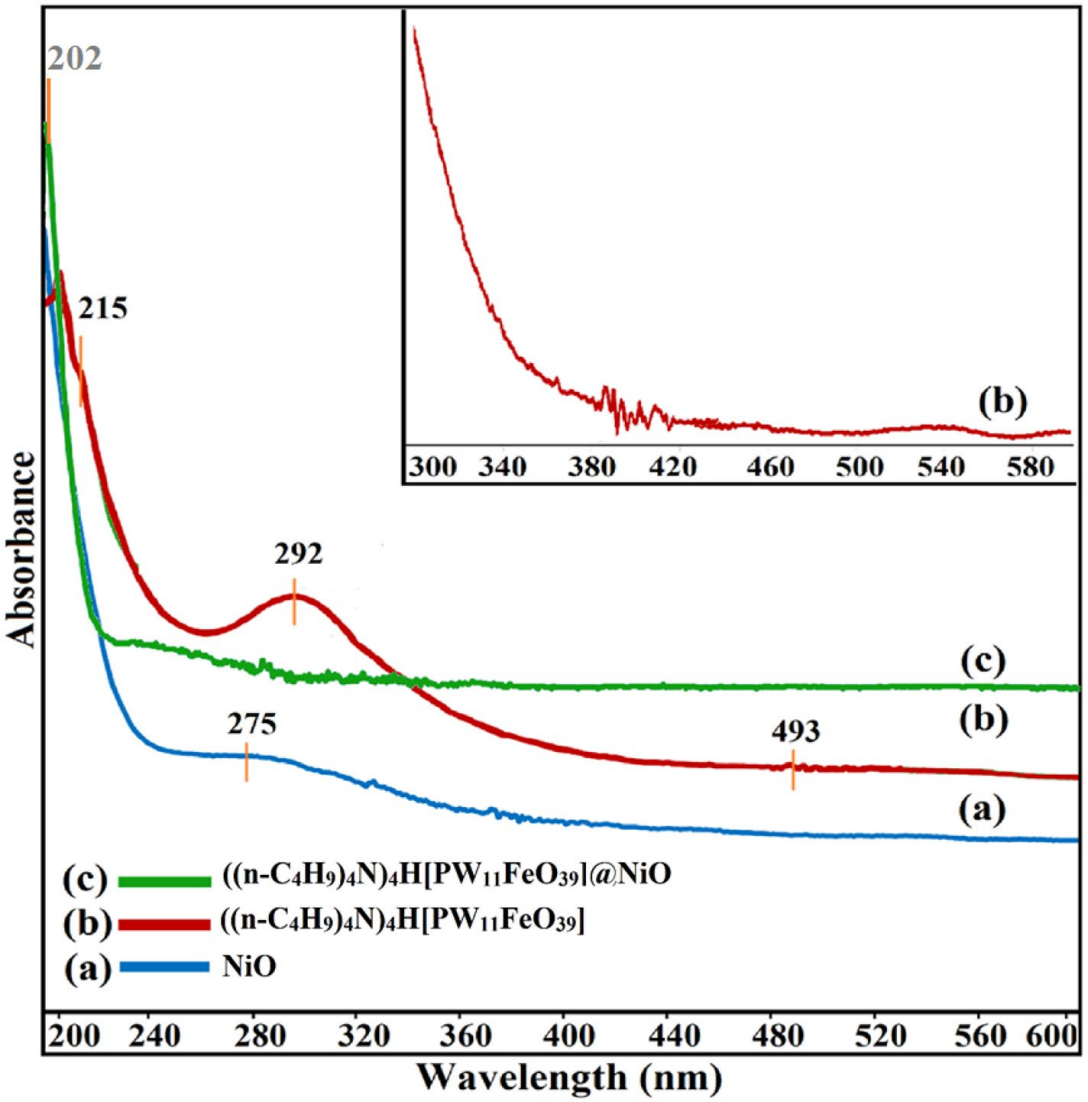
UV–Vis of (a) NiO nanoparticles, (b) ((n-C_4_H_9_)_4_N)_4_H[PW_11_FeO_39_], and (c) ((n-C_4_H_9_)_4_N)_4_H[PW_11_FeO_39_]@NiO).

For measuring the amount of light absorbed by the samples, UV–Vis spectroscopy was employed as a quantitative method. The UV–Vis spectra of (a) NiO nanoparticles, (b) (((n-C_4_H_9_)_4_N)_4_H[PW_11_FeO_39_], (c) and (((n-C_4_H_9_)_4_N)_4_H[PW_11_FeO_39_]@NiO were depicted in the range of 190–790 nm at ambient temperature (Fig. 5). Figure 5a demonstrates the absorption band of NiO nanoceramics in the range of 260–340 nm due to the ligand-to-metal charge-transfer (LMCT) transitions of oxygen to nickel (O^2-^ → Ni^2+^)^37,38^. Most characteristic peaks of the POMs are in the range of 200–300 nm. As indicated in Fig. 5b, the Keggin-type phosphotungstoferrate spectrum displays the principal bands at 215 and 292 nm, which are allocated to LMCT of tetrahedral oxygen to phosphorus (Oa^2−^ → P^5+^) and bridge oxygen to tungsten (O_b/c_^2−^ → W^6+^), respectively^39^. Moreover, the small broad peak at 493 nm is assigned to the d–d transitions of Fe^3+^^40^. The absorption peaks around the 198–200 nm wavelengths serve as proof for the πp-πd electronic transitions of the terminal bond (W = Od)^41^. Additionally, the absorption peaks of the ((n-C_4_H_9_)_4_N)_4_H[PW_11_FeO_39_]@NiO) nanocatalyst indicated that the coordination of the d-block metal elements in the Keggin structure and organic chain caused a hypochromic shift (absorption of shorter wavelengths by the molecule) (Fig. 5c). Furthermore, the characteristic bands of the ((n-C_4_H_9_)_4_N)_4_H[PW_11_FeO_39_]@NiO can be observed with substantial red-shift compared to the phosphotungstoferrate anions spectrum (202 nm → 215 nm). Considering the lower amount of ((n-C_4_H_9_)_4_N)_4_H[PW_11_FeO_39_] loading on NiO in the synthesis of nanocomposite, the characteristic band of polyoxometalate is not dominant compared NiO and the nanocomposite peak is similar to the NiO substrate. Therefore, the small peak corresponding to 492 nm, which is related to d–d transitions of Fe^3+^, is not observed in the nanocomposite spectrum. These findings can support the interactions between ((n-C_4_H_9_)_4_N)_4_H[PW_11_FeO_39_] and NiO substrate.

**Figure 6:**
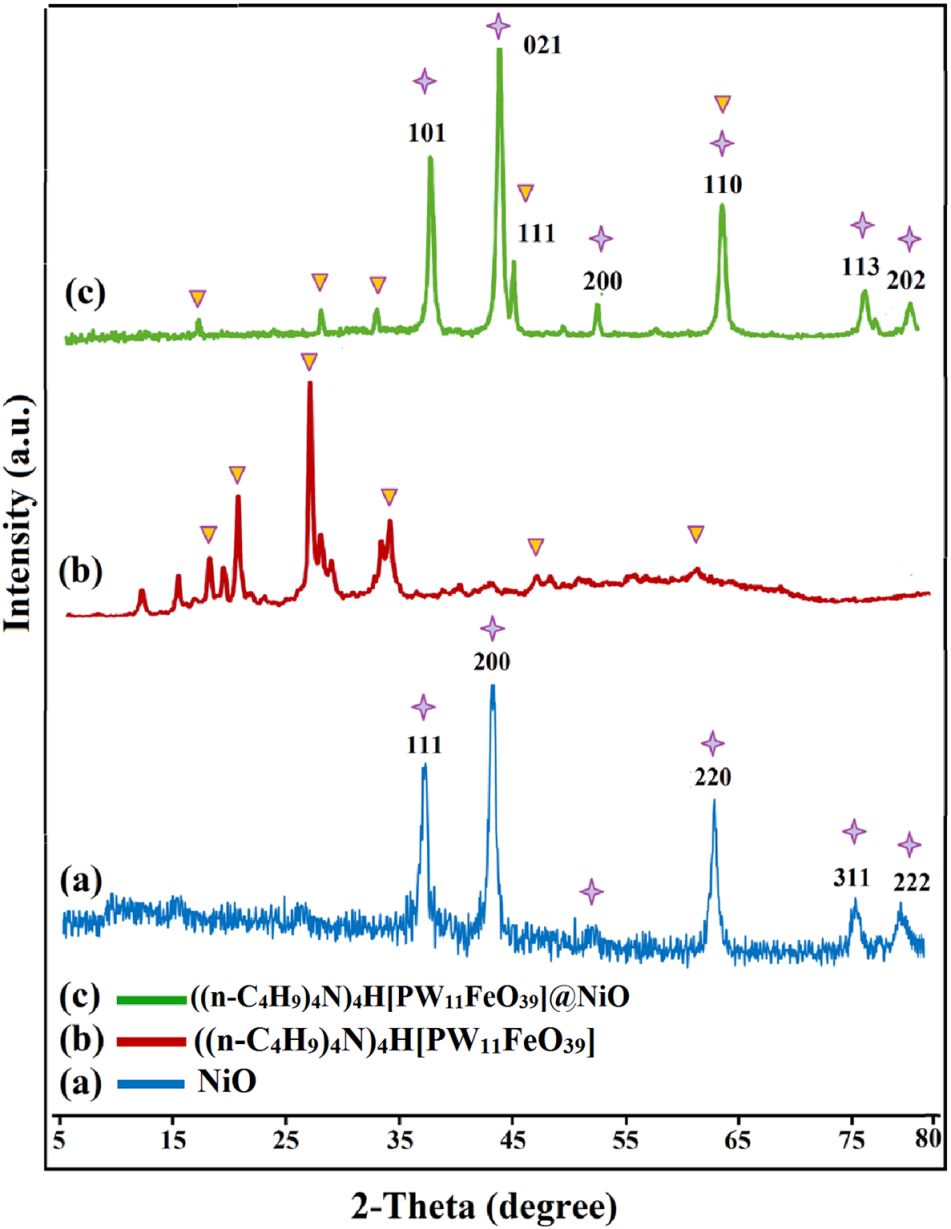
X-ray diffraction profiles of (a) NiO nanoparticles, (b) ((n-C_4_H_9_)_4_N)_4_H[PW_11_FeO_39_], and (c) ((n-C_4_H_9_)_4_N)_4_H[PW_11_FeO_39_]@NiO.

In order to analyze the phase and determine the size of grains and nanoparticles, the XRD technique was used (scanning range of $5^\circ \le 2\uptheta \le 80^\circ$). The diffraction patterns of (a) NiO nanoparticles, (b) ((n-C_4_H_9_)_4_N)_4_H[PW_11_FeO_39_] and (c) ((n-C_4_H_9_)_4_N)_4_H[PW_11_FeO_39_]@NiO are clearly shown in Fig. 6. According to Fig. 6a, the severe diffraction peaks at 2θ values of 36.4, 44.2, 63.9, 74.2, and 78.16° are attributed to the (101), (110), (113), and (202) planes of the face-centered cubic crystalline structure of NiO nanoparticles, respectively (JCPDS No. 04-0835)^42^. The XRD pattern of pure ((n-C_4_H_9_)_4_N)_4_H[PW_11_FeO_39_] depicts sharp peaks at 2θ values of 15.11, 18.00, 21.56, 21.80, 22.75, 29.60, 30.63, 32.42, and 34.58° as indexed with the JCPDS 00-050-0654 (Fig. 6b)^43^. The XRD of the desired nanocomposite is comprised primarily of NiO diffraction peaks, whereas the peaks with less intensity in the range of 15° to 30° depicted the presence of TBAPW_11_Fe species (Fig. 6c). The average size of the ((n-C_4_H_9_)_4_N)_4_H[PW_11_FeO_39_]@NiO crystal was computed by the Debye–Scherrer formula in the following manner^44^:

2 $${\text{D = }}\frac{{{\text{k}}\lambda \, }}{{\beta_{D} {\text{cos}}\theta }}$$

where D is the nanocrystalline size, k is the Scherrer constant (0.94), λ is the XRD radiation of wavelength (0.15406 nm), β is the full width at half maximum of peaks, and θ is the angle of reflection. By this formula [Eq. (2)], the average crystal size of the NiO, ((n-C_4_H_9_)_4_N)_4_H[PW_11_FeO_39_], and ((n-C_4_H_9_)_4_N)_4_H[PW_11_FeO_39_]@NiO nanoparticles was determined to be approximately 15.5, 48.3, and 28.1 nm, respectively.

**Figure 7:**
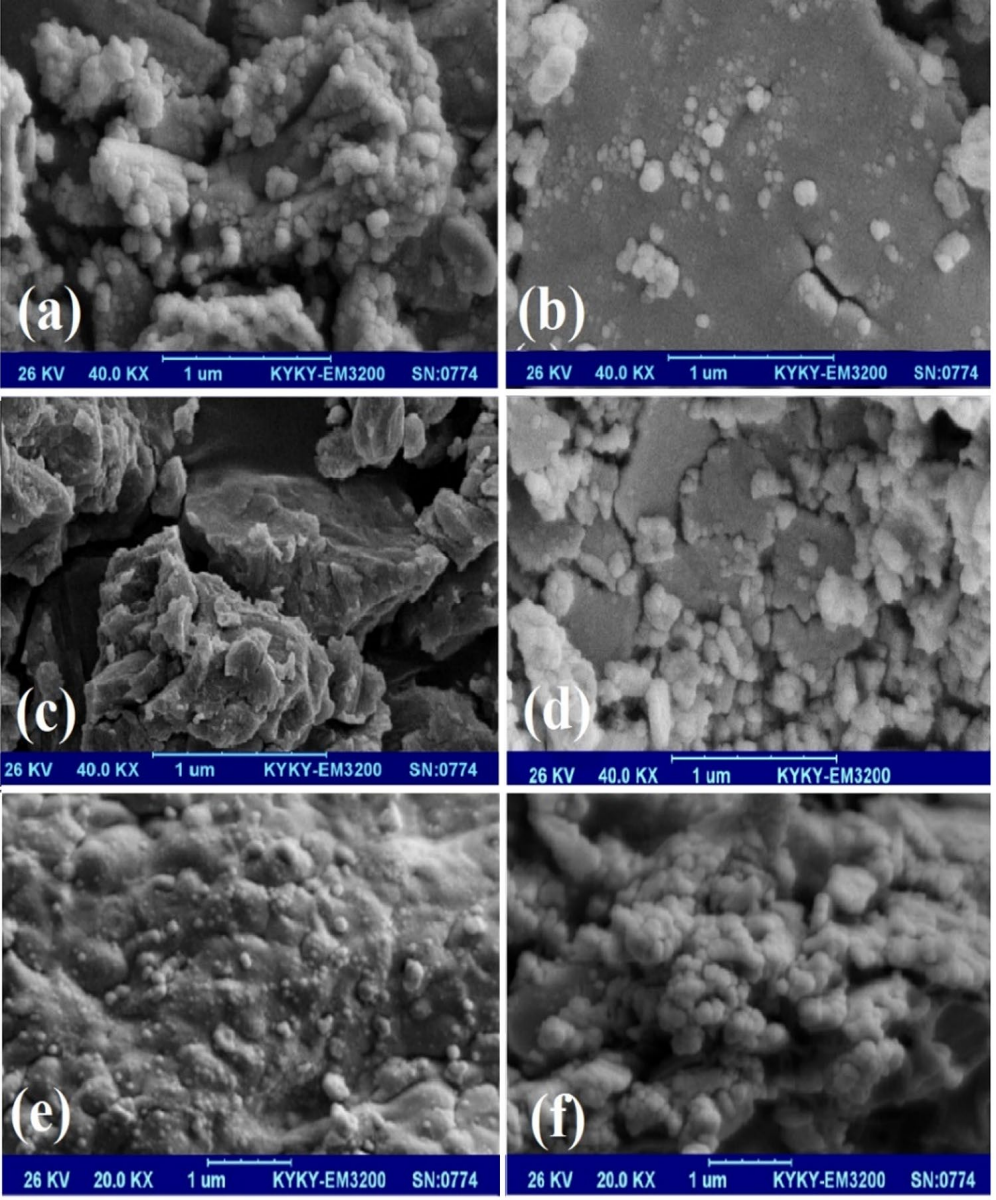
SEM images of (**a**, **b**) NiO nanoparticles (scale bar: 1 μm; magnification: 40.0 kx), (**c**, **d**) ((n-C_4_H_9_)_4_N)_4_H[PW_11_FeO_39_] (scale bar: 1 μm; magnification: 40.0 kx), and (**e**, **f**) ((n-C_4_H_9_)_4_N)_4_H[PW_11_FeO_39_]@NiO nanocatalyst (scale bar: 1 μm; magnification: 20.0 kx).

The morphological characteristics of the nanomaterials were investigated via SEM analysis, which is presented in Fig. 7. As depicted in Fig. 7a and b, it can be found that the NiO nanoparticles are in spherical shape and snowflake-like morphology due to the agglomeration process^45,46^. In the case of ((n-C_4_H_9_)_4_N)_4_H[PW_11_FeO_39_] (Fig. 7c,d), it is clear that the ((n-C_4_H_9_)_4_N)_4_H[PW_11_FeO_39_] nanoparticles are located on a rugged surface with an irregular and aggregated shape^45,46^. According to the Fig. 7e and f, the surface morphology of the ((n-C_4_H_9_)_4_N)_4_H[PW_11_FeO_39_]@NiO nanocatalyst indicates that the ((n-C_4_H_9_)_4_N)_4_H[PW_11_FeO_39_] spherical particles were placed on the rugged surface of the NiO with a large number of cavities. Each cavity is a suitable site for trapping the organosulfur compounds.

**Figure 8:**
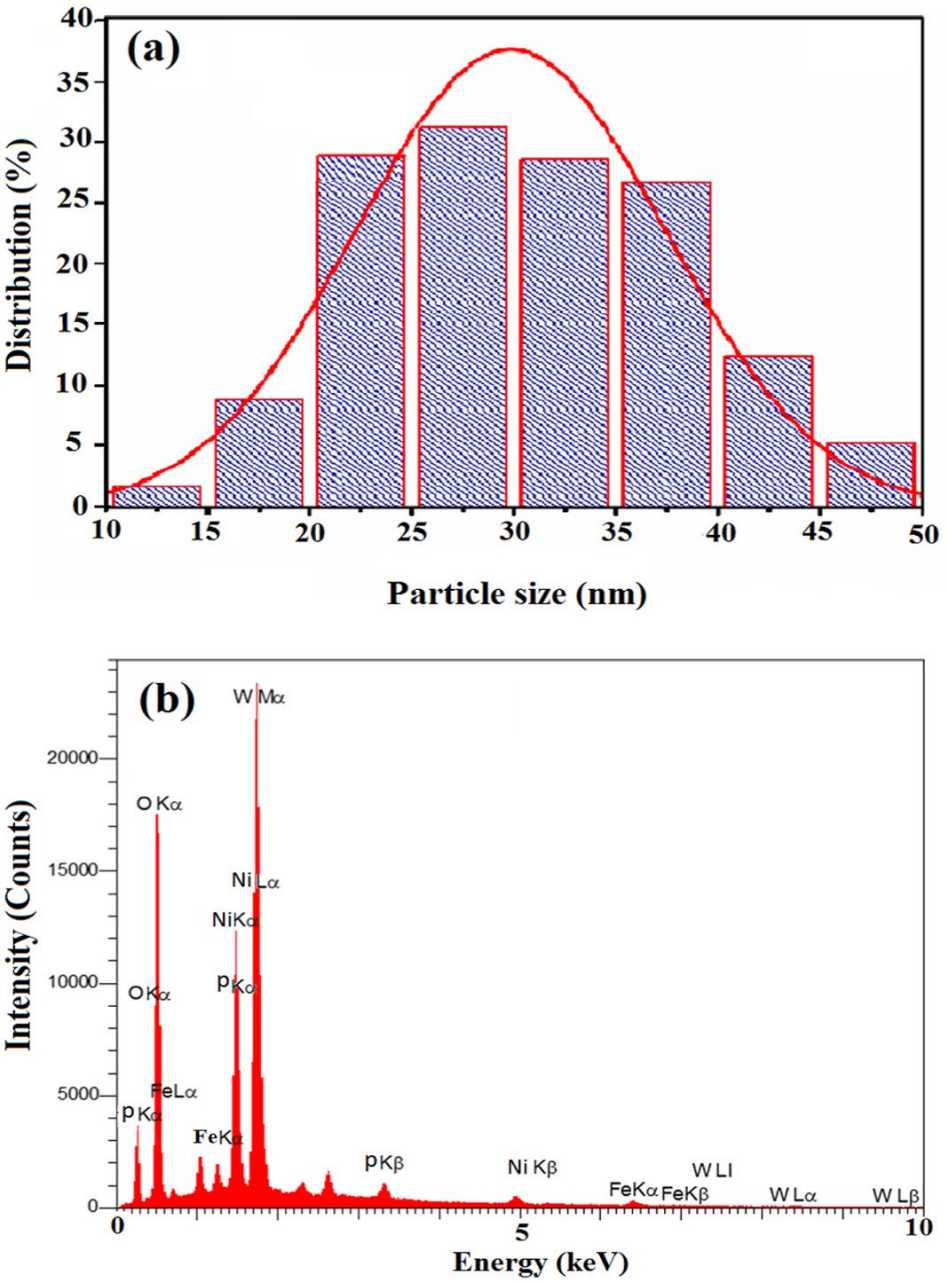
(**a**) EDX results of ((n-C_4_H_9_)_4_N)_4_H[PW_11_FeO_39_]@NiO, and (**b**) histogram diagram of the ((n-C_4_H_9_)_4_N)_4_H[PW_11_FeO_39_]@NiO nanocatalyst.

According to the histogram diagram provided in Fig. 8a, it can be inferred that the average particle size of the ((n-C_4_H_9_)_4_N)_4_H[PW_11_FeO_39_]@NiO falls within the spectrum of 25–30 nm. This finding is in close conformity with the particle size computed by Debye-Sherrer formula in the XRD outcomes (section “Results and discussion”). Moreover, the EDX analysis has confirmed the elemental composition percentage of the ((n-C_4_H_9_)_4_N)_4_H[PW_11_FeO_39_]@NiO nanocatalyst, as depicted in Fig. 8b. The EDX reveals the existence of O, W, Ni, Fe, and P atoms in the structure of nanocomposite. The approximate percentages of these elements found were 47.7, 29.4, 17.4, 4.5, and 0.9%, respectively.

**Figure 9:**
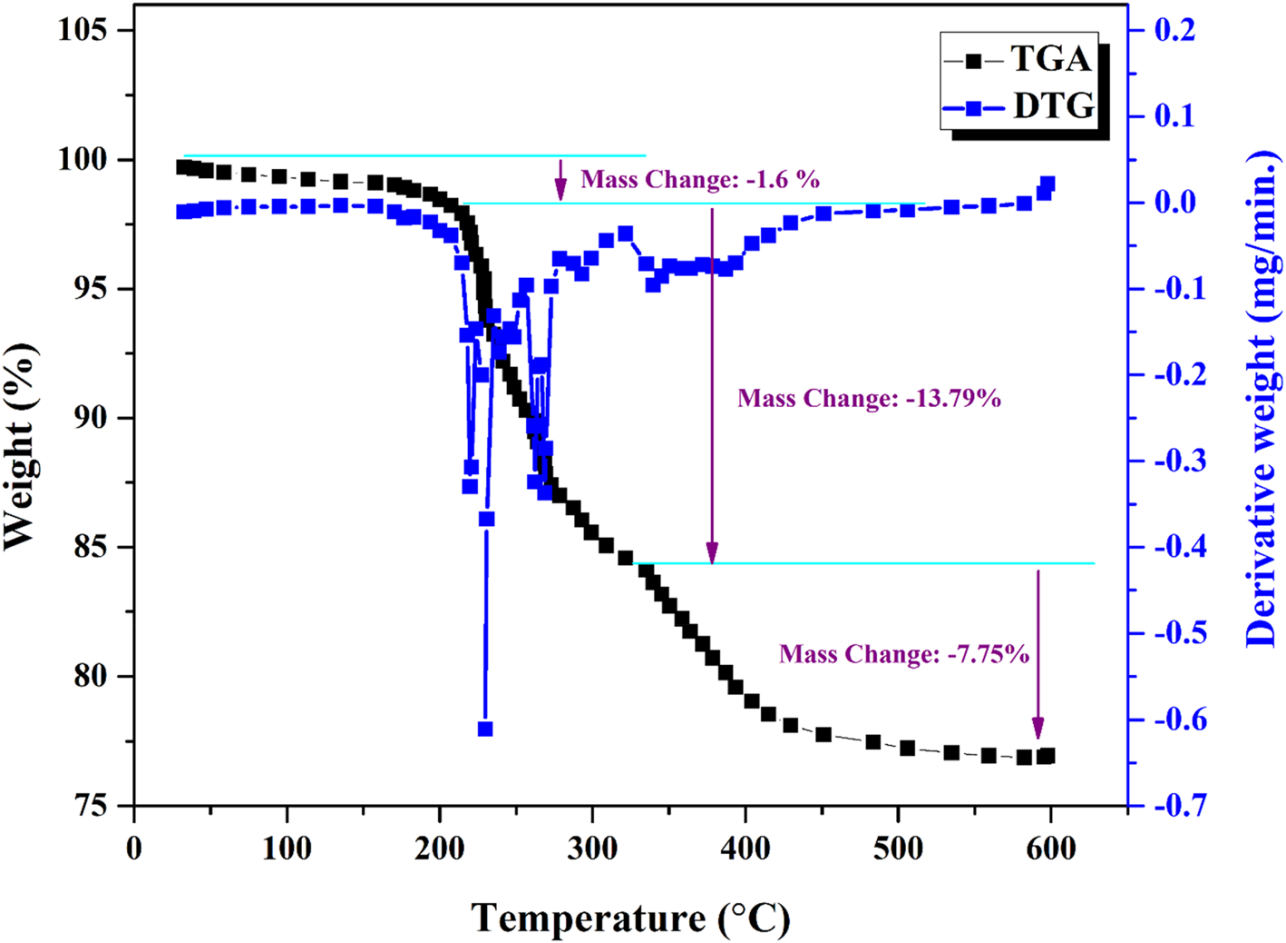
TGA–DTG of ((n-C_4_H_9_)_4_N)_4_H[PW_11_FeO_39_] from ambient temperature to 600 °C in a stream of flowing nitrogen.

Figure 9 exhibits the TGA-DTG evaluation of the (n-C_4_H_9_)4N)_4_H[PW_11_FeO_39_] while undergoing pyrolysis under N_2_ atmosphere. The TGA-DTG curves reveal the presence of multiple peaks, suggesting that the pyrolysis of POM involved several notable weight reduction processes. A first mass loss was observed up to 200 °C, which could be attributed to the loss of weakly bound water molecules from the polyoxometalate structure. In following, a second mass reduction occurs within the temperature range of 200 to 320 °C, which is attributed to the removal of structural crystalline water molecules from the (n-C_4_H_9_)4N)_4_H[PW_11_FeO_39_] unit. In the third stage, mass reduction up to 600 °C related to tetra butyl ions and oxygen decomposition of polyoxometalate was observed.

### ECOD results of real gas oil with facile extraction using MeCN

A typical procedure for the ECOD of real and model gas oil is described in the experimental part (Section “Preparation of ((n-C_4_H_9_)_4_N)4H[PW_11_FeO_39_]@NiO nanocomposite”). The main objective of this study is to identify and reduce the total S concentration of the real/model fuel using ((n-C_4_H_9_)_4_N)_4_H[PW_11_FeO_39_]@NiO nanocatalyst and acetic acid/H_2_O_2_ as an oxidizing agent. Thus, other characteristics related to gas oil properties, such as density (ASTM D-1298, g mL^−1^), water content (ASTM D-4006, vol%), salt content (ASTM D-3230, PTB), distillation (ASTM D-86, vol%), color and pouring remained unchanged after the ECOD treatment. The catalytic effectiveness of the ((n-C_4_H_9_)_4_N)_4_H[PW_11_FeO_39_]@NiO nanocatalyst was evaluated in the ECOD procedure by drastically reducing sulfur compounds from real gas oil. Some properties of the real gas oil are presented in Table 1 before and after the ECOD process. Table 1 shows that the amount of sulfur content decreased from 0.8735 to 0.0135 wt%, and the concentration of mercaptan (ppm) reduced from 265 to 6 ppm as well (Entries 1 and 2). As a result, the ((n-C_4_H_9_)_4_N)_4_H[PW_11_FeO_39_]@NiO nanocatalyst was highly influential in the removal of sulfur-containing compounds.

**Table 1:** The ECOD of real gas oil by ((n-C_4_H_9_)_4_N)_4_H[PW_11_FeO_39_]@NiO nanocatalyst.

Entry	Features of rea gas oil	Before ECOD	After ECOD^a^	After ECOD^b^
1	Total sulfur content wt%	0.8735	0.0301	0.0303
2	Density @ 15 °C	0.8248	0.8246	0.8246
3	Mercaptans ppm	265	6	7
4	Flash point (°F)	139	138	138
5	Water content vol%	0.024	0.024	0.024
6	Cloud point (°C)	− 4	− 4	− 4
7	Color test	1.6	1.6	1.6
8	Viscosity KIN @ 50 °C. CST	2.8	2.7	2.7
9	Pour point (°C)	− 10	− 10	− 10
10	Distillation (IBP °C)	160.5	160.3	160.4
11	Distillation (FBP °C)	386.4	386.2	386.1

^a^ECOD experiment condition: real gas oil (50 mL), ((n-C_4_H_9_)_4_N)_4_H[PW_11_FeO_39_]@NiO nanocatalyst (0.1 g), acetic acid-H_2_O_2_ (6 mL), extraction solvent (6 mL), time = 2 h, and temperature = 60 °C.

^b^ECOD of real gas oil by reused TBPW_11_Fe@NiO nanocatalyst.

### Effect of different catalysts on the ECOD of real/model gas oil

To study the catalytic capability of the ((n-C_4_H_9_)_4_N)_4_H[PW_11_FeO_39_]@NiO nanocatalyst, the reactivity trend of different heteropolyanion-based catalysts, including Keggin, Wells–Dawson and Preyssler, was investigated in the ECOD process under similar conditions (reaction temperature = 60 °C, reaction time = 120 min) (Table 2). The results indicated that the reactivity trend of the prepared thiophenic model fuels was reduced according to the following sequence: DBT > 4,6-DMDBT > 4-MDBT > BT. Among the various sulfur containing compounds used, DBT demonstrated high oxidative reactivity with an efficiency of 97%. The partial charge of electrons surrounded the S atom, and some steric hindrances can be assumed to affect the reactivity of thiophene molecules^47^. Among the different catalysts, the most outstanding efficiency was related to the ((n-C_4_H_9_)_4_N)_4_H[PW_11_FeO_39_]@NiO due to the presence of an organic chain in its structure and having a metal oxide substrate, and as a result, increasing the active surface of the catalyst (Entry 1 of Table 2). It is worthwhile to mention that the removal efficiency for DBT, 4.6-DMDBT, 4-MDBT, BT, and real fuel without nanocatalyst (blank test) was achieved 20, 18, 19, 18, and 17%, respectively (Entry 9). Following the introduction of the ((n-C_4_H_9_)_4_N)_4_H[PW_11_FeO_39_]@NiO, the ECOD efficiency has increased considerably up to 95% (Entry 1).

**Table 2:** Effect of different catalysts on the ECOD of real/model gas oil.

Entry	Catalyst	Conversion %					Ref.
		DBT	4-MDBT	4,6-DMDBT	BT	Gas oil	
1	TBPW_11_Fe@NiO	97	95	96	94	96	This work
2	TBPW_11_Fe	69	67	68	65	67	This work
3	NiO Nanoparticles	56	53	55	52	55	This work
4	(TBA)_4_PFeW_11_/PbO	97	94	95	93	97	^43^
5	PbO	58	55	57	53	55	^43^
6	H_14_[NaP_5_W_30_O_110_]	62	61	61	60	61	^48^
7	(Bu_4_N)_7_H_3_[P_2_W_18_Cd_4_]–TiO_2_	98	95.5	96	89	–	^48^
8	(Bu_4_N)_7_H_3_[P_2_W_18_Cd_4_]	92	90	91	84	–	^48^
9	Blank experiment	20	18	19	18	17	This work

ECOD experiment condition: real gas oil (50 mL), TBPW_11_Fe@NiO nanocatalyst (0.1 g), 6 mL of acetic acid-H_2_O_2_ (6 mL), 10 mL of MeCN solvent, time = 2 h, and temperature = 60 °C.

### Influence of ((n-C_4_H_9_)_4_N)_4_H[PW_11_FeO_39_]@NiO dosage on ECOD experiments

**Figure 10:**
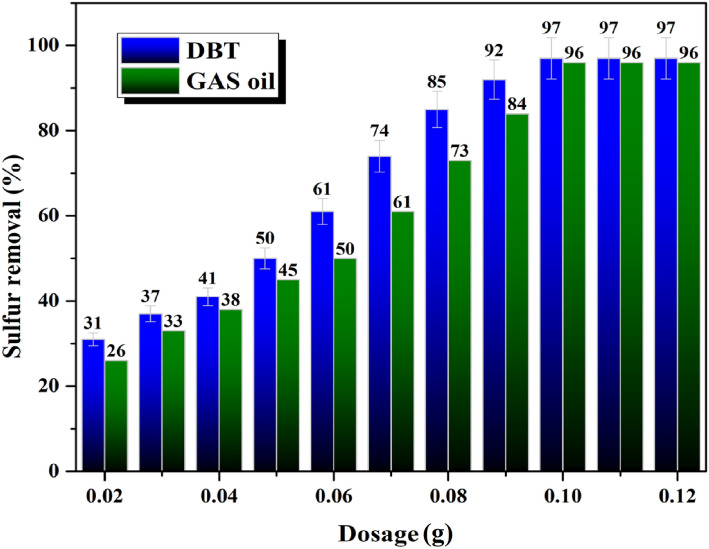
Effect of ((n-C_4_H_9_)_4_N)_4_H[PW_11_FeO_39_]@NiO dosage on elimination effectiveness of (a) real gas oil and (b) DBT.

From the ECOD process optimization perspectives, the ((n-C_4_H_9_)_4_N)_4_H[PW_11_FeO_39_]@NiO nanocatalyst dosage was assessed in the ECOD procedure. As pointed out in Fig. 10, the DBT and real gas oil desulfurization experiments were carried out by various amounts of ((n-C_4_H_9_)_4_N)_4_H[PW_11_FeO_39_]@NiO nanocatalyst (0.02–0.12 g). The desired amounts of the ((n-C_4_H_9_)_4_N)_4_H[PW_11_FeO_39_]@NiO were added separately to the closed-flask containing DBT and real diesel (50 mL). Afterward, the resultant mixture was heated to the reaction temperature and magnetically stirred for 120 min. In similar conditions, 0.10 g of the ((n-C_4_H_9_)_4_N)_4_H[PW_11_FeO_39_]@NiO displayed high catalytic efficiency in the ECOD procedure. Furthermore, superior amounts of the ((n-C_4_H_9_)_4_N)_4_H[PW_11_FeO_39_]@NiO nanocatalyst dosage (> 0.1 g) failed to further improve the effectiveness of the reaction. The elimination yield of as-prepared DBT and actual gas oil was obtained 97% and 96% utilizing 0.10 g of the ((n-C_4_H_9_)_4_N)_4_H[PW_11_FeO_39_]@NiO nanocatalyst, respectively. Accordingly, 0.1 g of the ((n-C_4_H_9_)_4_N)_4_H[PW_11_FeO_39_]@NiO was opted as the optimal amount for the desulfurization tests.

### Effect of temperature and time on the ECOD experiments

**Figure 11:**
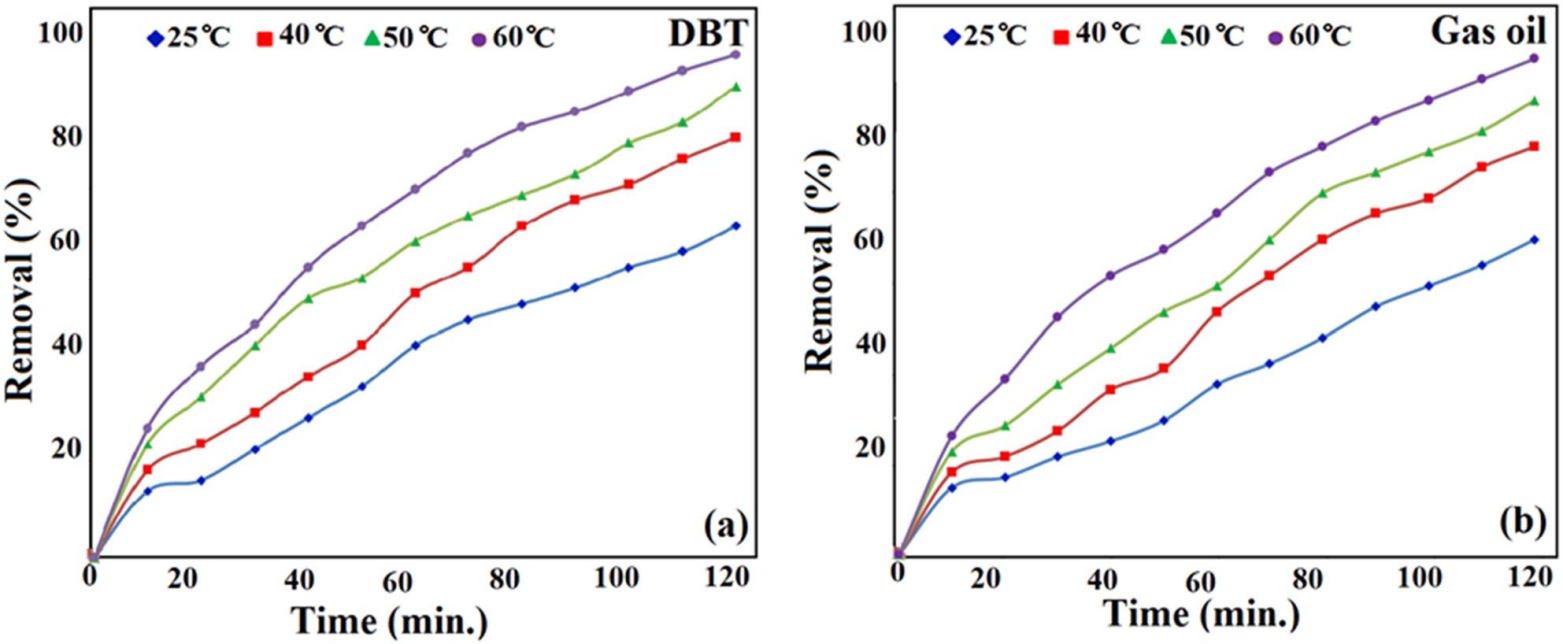
ECOD procedure for the catalytic desulfurization of (**a**) DBT and (**b**) real gas oil under different temperatures and times.

To further optimize the ECOD reactions, the process was carried out at the different times (0–120 min), and temperatures (25, 40, 50 and 60 °C). As shown in Fig. 11, the outcomes demonstrated that the change in temperature and time has a significant effect on the rate of the ECOD reactions, and the sulfur compounds were found to be eliminated when the temperature was increased. The sulfur concentration of DBT and actual fuel at 25 °C were diminished with a removal efficiency of 62 and 61%, respectively. When the treatment temperature was adjusted at 60 °C, the reaction performance (%) for DBT and real gas oil was reported 97 and 96%, respectively, after 2 h. Nevertheless, increasing the temperature rises up to 60 °C did not have a remarkable effect on the rate of the ECOD reactions. This may clarify the phenomena that the higher temperature ($\ge$ 60 °C) will cause the decomposition of H_2_O_2_, and the reduction of the concentration of W(O_2_)_n_ complex^49^. Thus, the reaction temperature and time were set at 60 °C and 2 h, respectively, as optimum conditions of the ECOD process.

### Kinetics study on the ECOD

**Figure 12:**
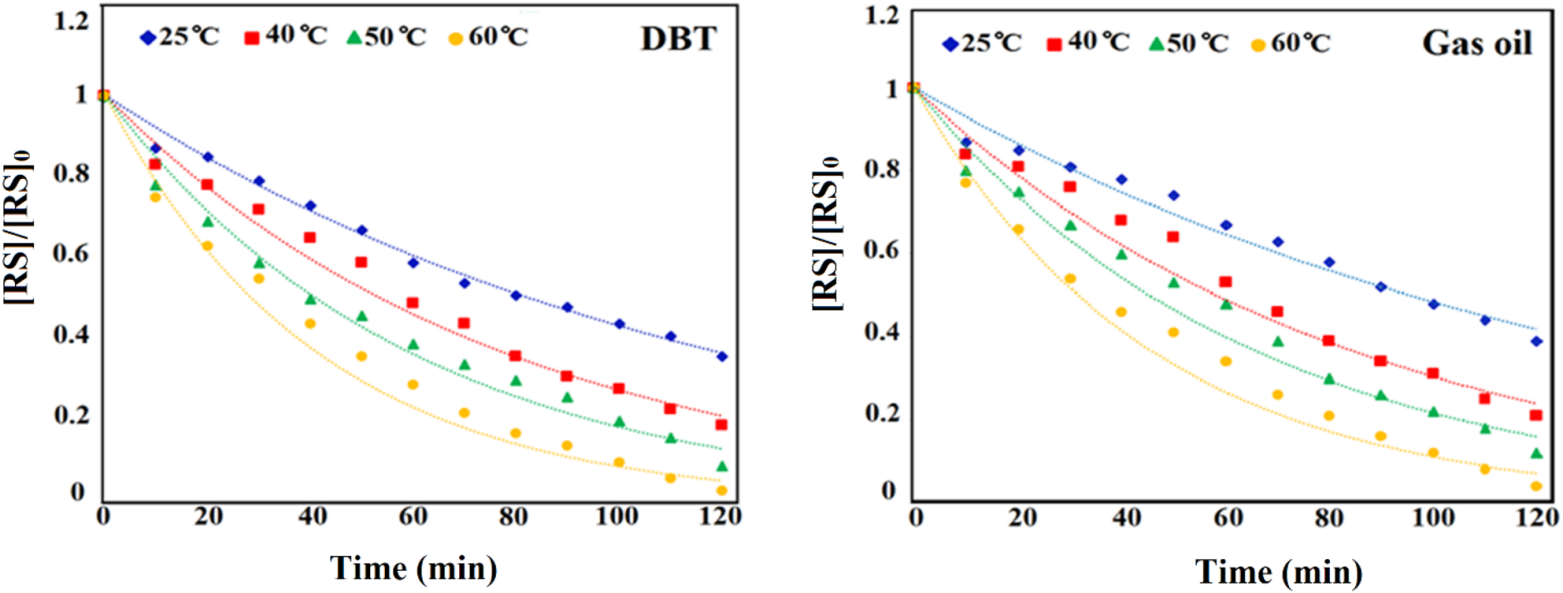
Curves of [RS]/[RS]_0_ vs. time (min) for the desulfurization procedure of real gas oil and DBT.

At various time intervals, the ECOD reaction kinetics have been studied for further understanding of sulfur compounds (RS) oxidation. As illustrated in Fig. 12, the slope of $\left[RS\right]$/${\left[RS\right]}_{0}$ against time (t) is straight-lined, indicating that desulfurization procedure of the as-prepared DBT and real gas oil follows pseudo-first-order reaction kinetics. The pseudo-first-order reaction equation was employed to calculate the values of the reaction rate constant (*k*) and correlation coefficient (*R*^*2*^). The kinetic reaction parameters were calculated according to the following equations [Eqs. (3)–(6)]^50^

3 $${r}_{RS}=\frac{d[RS]}{dt}=-k[RS]$$

4 $${\int }_{{[RS]}_{0}}^{[RS]}\frac{d[RS]}{[RS]}=-{\int }_{0}^{t}kdt$$

5 $$\mathit{ln}[RS]-\mathit{ln}{[RS]}_{0}=-kt$$

6 $$\mathit{ln}\frac{[RS]}{{[RS]}_{0}}=-kt \to \frac{\left[RS\right]}{{\left[RS\right]}_{0}}={e}^{-kt} \to \left[RS\right]={\left[RS\right]}_{0}{e}^{-kt}$$

where ${[RS]}_{0}$ (mg/L) and $[RS]$ ($m$ g/L) are initial and t-moment (min) concentrations, respectively. The reaction rate constant of *K* (min^−1^) was calculated from slop of ln [RS]/[RS]_0_ according to the time in constant temperature.

Under the results of Table 3, the values of rate constant (min^−1^) and correlation coefficient (R^2^) were more significant than 0.90 ($\sim 1)$ at different temperatures (25, 40, 50 and 60 °C).

**Table 3:** Pseudo-first-order rate constants (K) and correlation factors (R^2^) of the ECOD.

Temperature (°C)	k (min^−1^)			R^2^
	Gas oil	Dibenzothiophene	Gas oil	Dibenzothiophene
25	0.031	0.08	0.9187	0.9940
40	0.035	0.13	0.8892	0.9838
50	0.055	0.17	0.9174	0.9581
60	0.060	0.24	0.9477	0.9517

**Figure 13:**
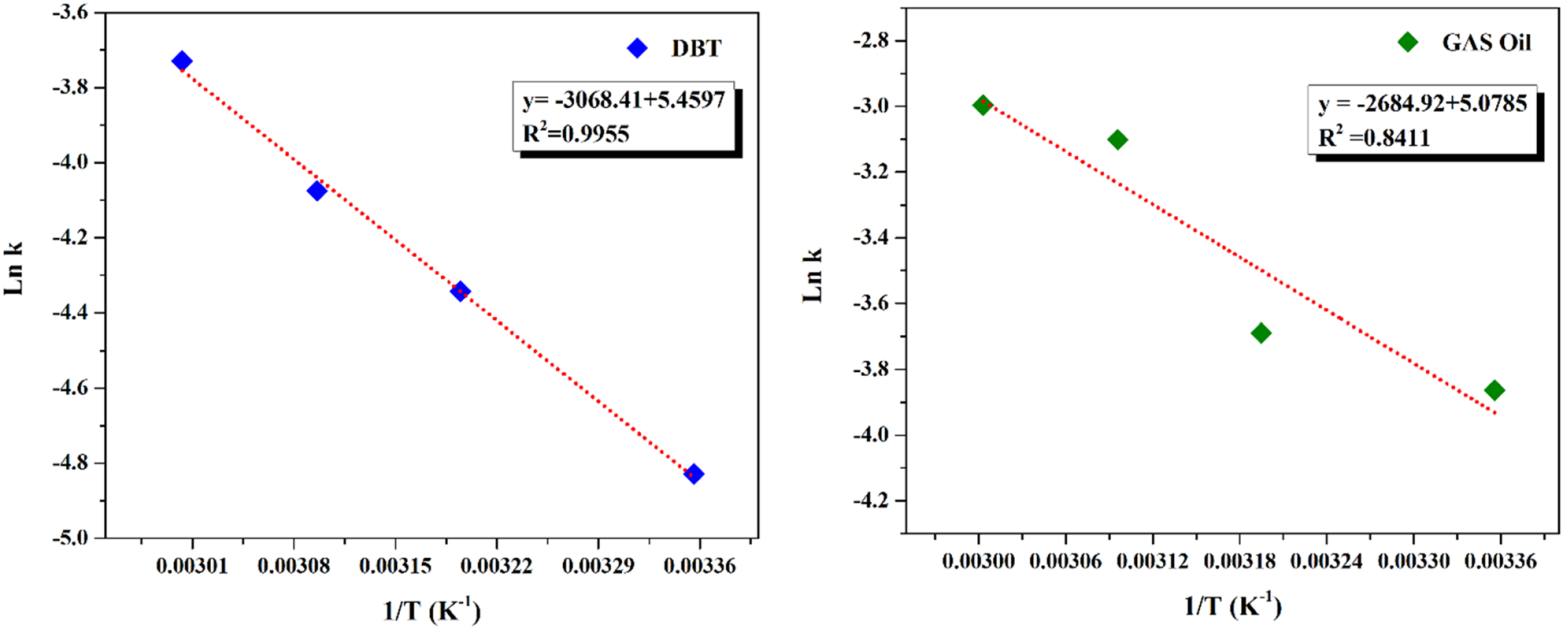
Arrhenius curves for the oxidation of model and real gas oil.

By determining the rate constant k over a variety of temperatures and then applying the Arrhenius equation [Eq. (7)], the activation energy (Ea) for the removal of sulfur molecules in the ECOD process was computed (Fig. 13)^50^. The Ea values for the desulfurization of the model and real gas oil were attained 22.73 and 22.32 kJ mol^−1^, respectively.

7 $$k=A exp(\frac{{-E}_{a}}{RT})$$

where *k* represents the rate constant, *E*_a_ is the activation energy, *R* is the gas constant ($\frac{8.3145{\text{J}}}{{\text{K \, mol}}}$), and *T* is the temperature expressed in Kelvin, *A* is known as the frequency factor, having units of L mol^−1^ s^−1^, and takes into account the frequency of reactions and the likelihood of correct molecular orientation^51^.

### Possible mechanism for the oxidation reaction of DBT

**Figure 14:**
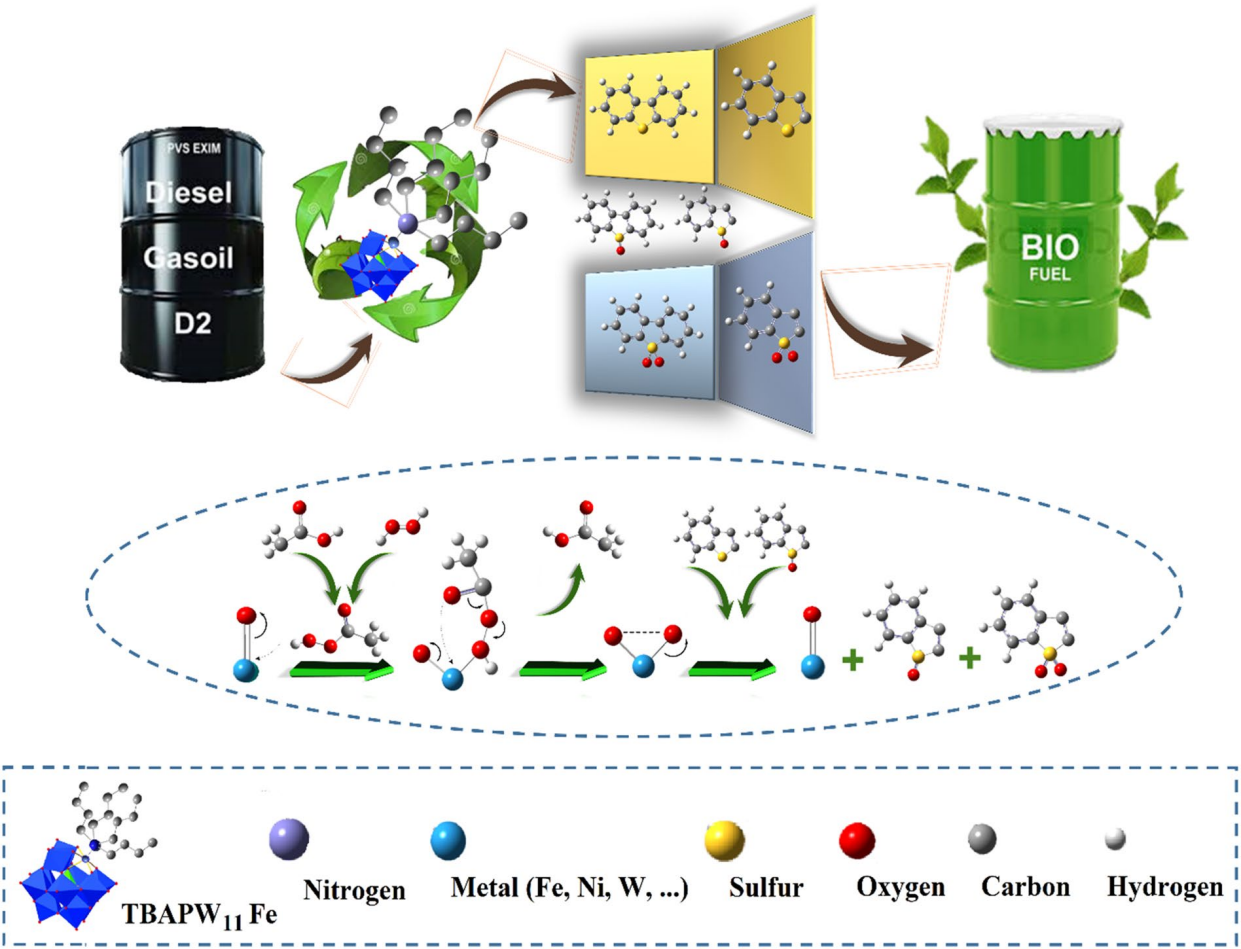
Schematic illustration of the suggested ECOD mechanism of prepared model gas oil catalyzed by ((n-C_4_H_9_)_4_N)_4_H[PW_11_FeO_39_]@NiO phase-transfer catalyst.

The following mechanism was suggested for the oxidation of model gas oil (DBT) by the ((n-C_4_H_9_)_4_N)_4_H[PW_11_FeO_39_]@NiO heterogeneous catalyst (M(O)^n−^ (M = Fe, W or Ni) (Fig. 14). In the onset of the reaction, the use of acetic acid/H_2_O_2_ as an oxidant system at a volume ratio of 1:2 results in the in-situ production of peroxy acid without forming of a significant amount of residual^52,53^. Afterward, peroxy acid reacts with the terminal oxygen (M = O_t_) of the VO_6_ octahedral unit of Keggin-type ((n-C_4_H_9_)_4_N)_4_H[PW_11_FeO_39_], leading to the formation of an intermediate peroxo-((n-C_4_H_9_)_4_N)_4_H[PW_11_FeO_39_](M(O_2_)). The sulfur containing compounds can attack the intermediate oxoperoxo species (M(O_2_))_n_ species to produce the corresponding sulfoxide and sulfones. The existence of Bu_4_N^+^Br^−^ as an organic tail and phosphotungstate as an aqueous part resulted in the formation of an amphiphilic substance that increases the catalyst efficiency and facilitates phase transition. Moreover, the NiO nanoceramics prevent the aggregation of ((n-C_4_H_9_)_4_N)_4_H[PW_11_FeO_39_] clusters, thereby increasing the active sites. Eventually, the corresponding products of the reaction (sulfone and sulfoxide) accumulate in the aqueous phase by acetonitrile (10 mL).

### Recycling performance of TBAPW_11_Fe@NiO in the ECOD process

**Figure 15:**
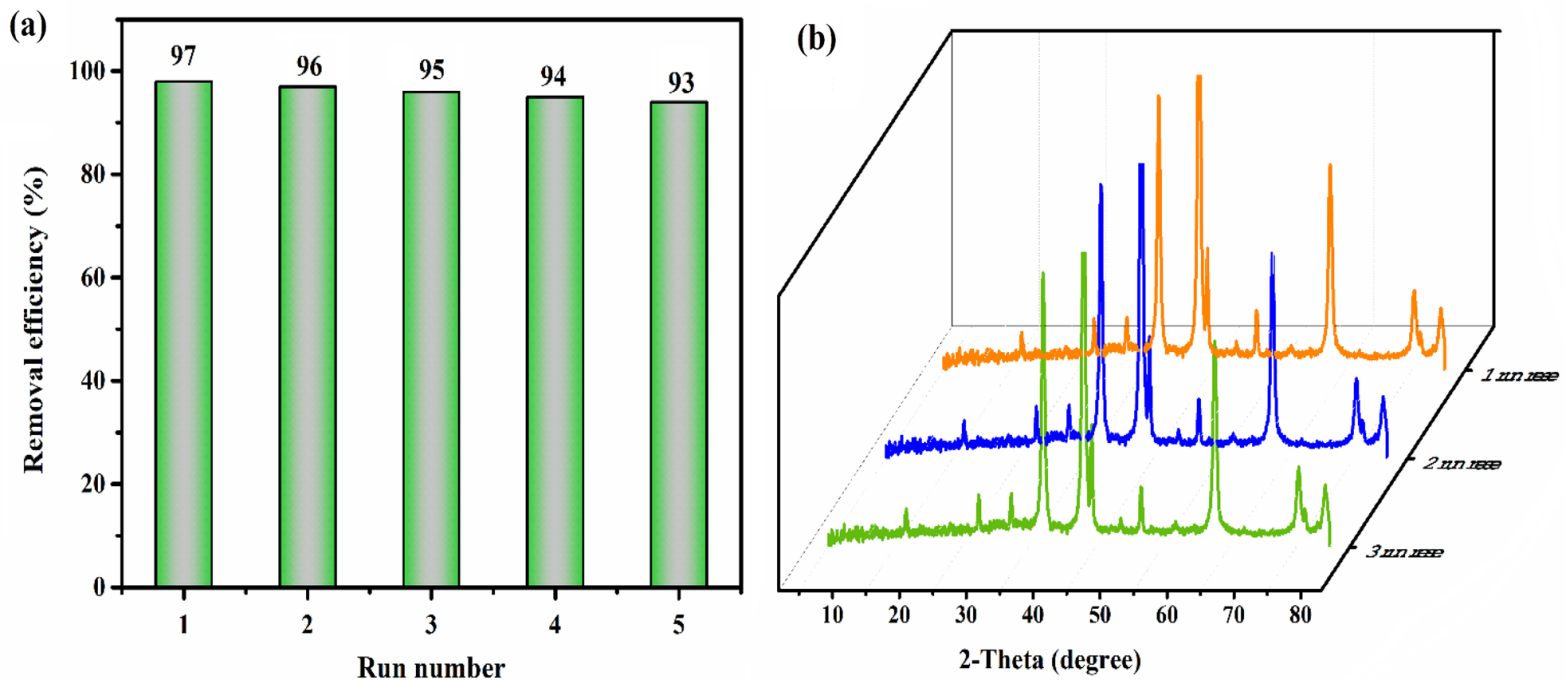
(**a**) Recyclability efficiency of the ((n-C_4_H_9_)_4_N)_4_H[PW_11_FeO_39_]@NiO in the ECOD process of as-prepared model gas oil (DBT), (**b**) XRD pattern of nanocatalyst after first, third, and fifth run reuse.

To find out the lifetime of any catalyst, it can be recovered and reused. In this regard, the recyclability of the (n-tBu)_4_N)_4_H[PW_11_FeO_39_]@NiO phase-transfer catalyst was investigated in the ECOD process of as-prepared model gas oil (DBT) (Fig. 15). After completing the reaction, the nanocatalyst was centrifuged and washed with dichloromethane (CH_2_Cl_2_). Afterward, it was dried at a temperature of 90 °C and applied to the subsequent process without further purification. The TBAPW_11_Fe@NiO heterogeneous catalyst could be reused five times without significantly diminishing its catalytic performance. The data provided in Fig. 15a demonstrated a gradual decline in reaction yield from 97 to 93% over five successive runs. Furthermore, The XRD pattern of the nanocatalyst after five cycles did not reveal any significant change compared to the primary structure (Fig. 15b).

## Conclusions

In this study, the fundamental requirement of cleaning the fuel by minimizing the S content in the range of international standards was successfully carried out by the ECOD procedure. To summarize, a new nanocomposite was prepared auspiciously by supporting Keggin-type phosphotungstoferrate (((n-C_4_H_9_)_4_N)_4_H[PW_11_FeO_39_]) on nickel(II) oxide nanoceramics as a heterogeneous phase transfer catalyst, and further employed in the ECOD process of model/real gas oil. The results of the characterization analyses confirmed that the materials were composed satisfactorily. The ECOD reactions of sulfur-containing compounds were catalyzed using H_2_O_2_/AcOH (volume ratio of 2:1) as an oxidant in the presence of the ((n-C_4_H_9_)_4_N)_4_H[PW_11_FeO_39_]@NiO nanocatalyst. Afterwards, a series of tests were conducted to determine the influences of catalyst dosage, time and temperature on the ECOD process. According to the experimental findings, the heterogeneous catalyst displayed outstanding catalytic performance (up to 95%) with 0.1 g at a temperature of 60 °C and contact time of 120 min. The ((n-C_4_H_9_)_4_N)_4_H[PW_11_FeO_39_]@NiO nanocatalyst can be recycled five times with a minor drop. Moreover, the oxidation process of the as-prepared DBT and real gas oil follows pseudo-first-order reaction kinetics. Finally, a mechanism involving oxo-peroxo intermediate species was proposed for the oxidation of prepared thiophenic model fuel (DBT) and real gas oil. This work provided valuable insights for developing practical phase transfer catalysts for the profound ODS process.

## Data Availability

The datasets used and/or analyzed during the current study available from the corresponding author on reasonable request.
